# A Systems Biology Approach to Reveal Putative Host-Derived Biomarkers of Periodontitis by Network Topology Characterization of MMP-REDOX/NO and Apoptosis Integrated Pathways

**DOI:** 10.3389/fcimb.2015.00102

**Published:** 2016-01-11

**Authors:** Fares Zeidán-Chuliá, Mervi Gürsoy, Ben-Hur Neves de Oliveira, Vural Özdemir, Eija Könönen, Ulvi K. Gürsoy

**Affiliations:** ^1^Programa de Pós-Graduação em Ciências Biológicas: Bioquímica, Departamento de Bioquímica, Instituto de Ciências Básicas da Saúde, Universidade Federal do Rio Grande do SulPorto Alegre, Brazil; ^2^Department of Periodontology, Institute of Dentistry, University of TurkuTurku, Finland; ^3^Faculty of Communications and Office of the President, International Technology and Innovation Policy, Gaziantep UniversityGaziantep, Turkey; ^4^Amrita School of Biotechnology, Amrita Vishwa Vidyapeetham (Amrita University)Kollam, India; ^5^Oral Health Care, Welfare DivisionTurku, Finland

**Keywords:** gelatinases, oxidative stress, saliva, drug target, omics, computational biology, *in silico*, network

## Abstract

Periodontitis, a formidable global health burden, is a common chronic disease that destroys tooth-supporting tissues. Biomarkers of the early phase of this progressive disease are of utmost importance for global health. In this context, saliva represents a non-invasive biosample. By using systems biology tools, we aimed to (1) identify an integrated interactome between matrix metalloproteinase (MMP)-REDOX/nitric oxide (NO) and apoptosis upstream pathways of periodontal inflammation, and (2) characterize the attendant topological network properties to uncover putative biomarkers to be tested in saliva from patients with periodontitis. Hence, we first generated a protein-protein network model of interactions (“BIOMARK” interactome) by using the STRING 10 database, a search tool for the retrieval of interacting genes/proteins, with “Experiments” and “Databases” as input options and a confidence score of 0.400. Second, we determined the centrality values (*closeness, stress, degree* or *connectivity*, and *betweenness*) for the “BIOMARK” members by using the Cytoscape software. We found Ubiquitin C (UBC), Jun proto-oncogene (JUN), and matrix metalloproteinase-14 (MMP14) as the most central hub- and non-hub-bottlenecks among the 211 genes/proteins of the whole interactome. We conclude that UBC, JUN, and MMP14 are likely an optimal candidate group of host-derived biomarkers, in combination with oral pathogenic bacteria-derived proteins, for detecting periodontitis at its early phase by using salivary samples from patients. These findings therefore have broader relevance for systems medicine in global health as well.

## Introduction

Periodontitis is a chronic infection with a progressive inflammatory process of the tooth-supporting tissues and characteristically causes gingival recession, alveolar bone loss, and mobility of the teeth. If the necessary treatment is not performed, this disease can lead to the loss of affected teeth. Several pathways, such as apoptosis, matrix metalloproteinase (MMP)-REDOX/nitric oxide (NO) activation, toll-like receptor, and nuclear factor-kB (NF-kB) signaling, cytokine and chemokine network, complement cascade, and osteoclastogenesis, play a role in the pathogenesis of this chronic disease with remissions and exacerbations (Cekici et al., 2014).

The two host cell types that mainly come across with periodontopathogenic bacteria during inflammation in periodontal tissues are epithelial cells and polymorphonuclear leukocytes (PMNL). The epithelium expresses cytokines, chemokines, proteases, and natural antimicrobial peptides against infectious stimuli (Gursoy and Könönen, 2012). Once bacteria bind to the PMNL surface, phagocytosis results in entrapment of the bacterial cell into a membrane-delimited structure, also known as phagosome. The phagosome undergoes maturation by fusion with endosomes and finally with lysosomes. Lysosomal vesicles include reactive oxygen species (ROS). ROS activate the redox sensitive NF-kB signaling pathway, which induces the expression of cell adhesion receptors, proinflammatory cytokines and chemokines, involved in the production of free radicals and persistence of inflammation (Scott and Krauss, 2012). On one hand, ROS destroy pathogenic bacteria and other phagocytosed material within the safe confines of the phagolysosome. On the other hand, the extracellular release of oxygen intermediates leads to significant destruction in periodontal tissues. This damage is mainly an outcome of elevated MMP activation and increased apoptosis of gingival resident cells (Zeidán-Chuliá et al., 2013, 2014a).

Biomarkers of periodontitis are sorely needed to intervene early in the course of the disease. Periodontitis causes little, if any, discomfort at its initial stages thereby allowing the clinical diagnosis not until alveolar bone loss has already commenced or materialized (Gursoy et al., 2011). Over the past decade, research on salivary diagnostics received attention by virtue of an easy sample access and low cost, while the search for biomarkers broadened in scope to characterize both the human host and resident bacteria using systems science and omics technology (e.g., genomics and/or proteomics) approaches (Cuevas-Córdoba and Santiago-García, 2014; Gürsoy et al., 2014).

It is now widely accepted that periodontitis is a consequence of complex host-environment and biological pathway interactions, rather than a product of a single gene or protein (Hajishengallis and Sahingur, 2014). Network modeling by systems biology-based approaches is increasingly employed as versatile effective tools to unravel the pathogenesis of periodontal disease by integration of multi-omics data (Zhu et al., 2007; Zeidán-Chuliá et al., 2013, 2014a; Gürsoy et al., 2014). Moreover, by studying the topological network properties within an interactome, one might identify nodes (e.g., genes/proteins) with a biologically critical position (bottlenecks) in the overall network architecture and thus putative early disease biomarkers and drug targets. In general, the word “bottleneck” refers to nodes with high *betweenness* values, indicating that those nodes are central points that control the communication between other nodes within the network. These nodes are “between” highly interconnected subgraph clusters and by removing them, the network could be divided (Yu et al., 2007).

In previous studies, we reported the deregulated expression of apoptosis and MMP-REDOX/NO-related genes in periodontitis samples when compared to those of healthy controls (Zeidán-Chuliá et al., 2013, 2014a). Most common histological findings in early periodontitis are related to neutrophil migration and activation and weakened wound healing in resident cells (Biasi et al., 1999). Neutrophils are one main source of tissue degrading MMPs, while oxidative stress-induced apoptosis of resident cells (gingival epithelia and fibroblasts) is one main outcome of increased bacterial invasion and decreased tissue regeneration (Nussbaum and Shapira, 2011). Therefore, our aims for the present study were (1) to simultaneously analyze these two initial pathways of periodontal inflammation (MMP-REDOX/NO and apoptosis) by systems biology, and (2) to define their functional interconnections as putative biomarkers of early periodontitis.

## Materials and methods

### Interaction network development, analysis of topological network properties and landscape visualization of centrality values

The “BIOMARK” interactome was developed by using the STRING 10 database (http://string-db.org/; Szklarczyk et al., 2011, 2015) with “Experiments” and “Databases” as input options and a confidence score of 0.400. STRING is a search tool for the retrieval of interacting genes/proteins extracted from diverse curate and public databases with information on direct and indirect functional associations/interactions. Interactions are derived from different sources (1) primary databases, (2) manually-curated databases, (3) Medline abstracts and a large collection of full-text articles, (4) algorithms and co-expression analysis using genomic information, and (5) interactions observed in one organism that are systematically transferred to others via pre-computed orthology relations (Szklarczyk et al., 2015).

**Figure 1:**
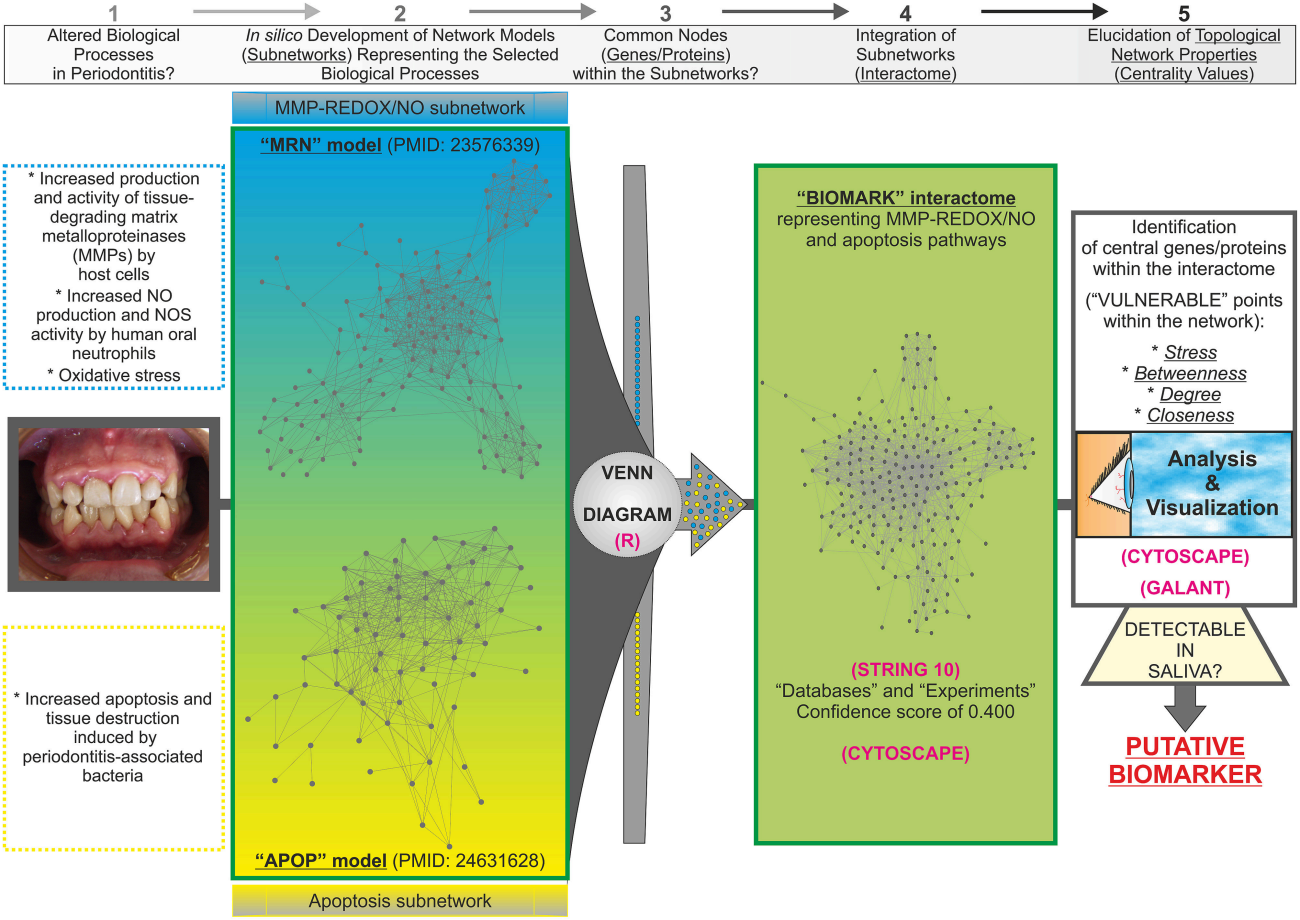
**Abstract workflow summarizing the criteria and tools used for the present study**. The “MRN” and “APOP” *in silico* models, already available in the literature, were used as the starting material to develop the “BIOMARK” integrating MMP-REDOX/NO and apoptosis related-genes/proteins into one single network of interactions. The Venn diagram, performed by using the software system R, revealed the existence of common nodes (genes/proteins). Thereafter, the interactome was developed for integrating these subnetworks into one single gene/protein interaction network model (“BIOMARK”) by using the STRING 10 and Cytoscape tools. Further elucidation of the topological network properties reveals “vulnerable” points within the *in silico* model representing candidate diagnostic biomarkers and also potential molecular targets for therapeutics. With GALANT, a Cytoscape plugin, we project numerical data from the topological network properties (centrality measurements) onto “BIOMARK” interactome to create a smoothed data map resembling the network layout. Central members of “BIOMARK,” easily detectable in saliva, represent optimal candidates to be tested as periodontitis biomarkers.

As a starting point, we selected two published *in silico* network models to get the list of genes/proteins that would be part of the interactome (Figure 1): the “MRN” model with MMP and REDOX/NO-related genes/proteins (Zeidán-Chuliá et al., 2013) and the “APOP” model with apoptosis-related genes/proteins (Zeidán-Chuliá et al., 2014a). The criteria to select these models (subnetworks) were based on biological processes typically altered in periodontitis, such as (1) increased production and activity of MMPs by host cells, (2) increased NO production and NOS activity by human oral neutrophils, (3) oxidative stress, as well as (4) increased apoptosis and tissue destruction induced by periodontal pathogens. *In silico* integration of the two subnetworks onto one interactome would characterize above-mentioned biological processes at the molecular level for the search of potential biomarkers of periodontitis. Thereafter, a Venn diagram was constructed by using the freely available software system R (http://www.r-project.org; Gentleman et al., 2004) in order to visualize the grade of molecular relation (common genes/proteins) between the “MRN” and “APOP” subnetworks. The genes/proteins that integrated the “BIOMARK” interactome were identified by using the Human Genome Organization (HUGO) Gene Symbol (Wain et al., 2004) and Ensembl protein ID (Birney et al., 2006). The selected list (Supplementary Table 1) was applied into the STRING database and the links (interaction strength) between two different nodes (genes/proteins) were saved in data files and handled by utilizing the Cytoscape open source software platform. Cytoscape is used for visualizing complex networks and integrating these with any type of attribute data (Smoot et al., 2011). The original Cys file of “BIOMARK” model is additionally provided as Supplementary Material (Supplementary Data Sheet 1). Topological network properties (Yu et al., 2007) such as *closeness, stress, degree* or *connectivity*, and *betweenness* centralities (Supplementary Table 2) were also analyzed by using the NetworkAnalyzer plugin from the Cytoscape software. Values of centralities above one standard deviation (+1 SD) of the mean were selected to identify potential candidate host-derived biomarker/s.

Of note, *closeness* measures the grade of proximity of a node to the rest of nodes. The larger the value, the faster the information spreads through this node. *Stress* measures the number of times a given node is traversed by ideal routes or “shortest paths” within a network. Nodes that are traversed by higher numbers of short paths are by definition more stressed. *Degree* measures the local topology of each node by summing up the number of its adjacent nodes. Nodes with high values of *degree* over the thresholds values are named as “hubs.” *Betweenness*, which is similar to *stress* as a topological network property, measures how frequently the shortest path, connecting every pair of nodes, is going through a third given node. Therefore, both *stress* and *betweenness* provide information about the influence of a node over the spread of information throughout the interactome. All nodes with high values of *betweenness* over the thresholds values were named as “bottlenecks” (non-hub- and hub-bottlenecks are represented by NH-B and HB, respectively).

For the network-level visualization of centrality values in the “BIOMARK” interactome, we utilized GALANT (GrAph LANdscape VisualizaTion). GALANT is a Cytoscape plugin that builds functional landscapes onto biological networks (Camilo et al., 2013). GALANT projects any kind of numerical data (e.g., centrality measurements) onto a network in order to create landscapes resembling the network layout, and also offers a friendly interface fully integrated with the Cytoscape where users can easily build their landscapes of interest by using one of the previously mentioned functions.

## Results

### “BIOMARK” is an integrative network model for MMP-REDOX/NO and apoptosis subnetworks

**Figure 2:**
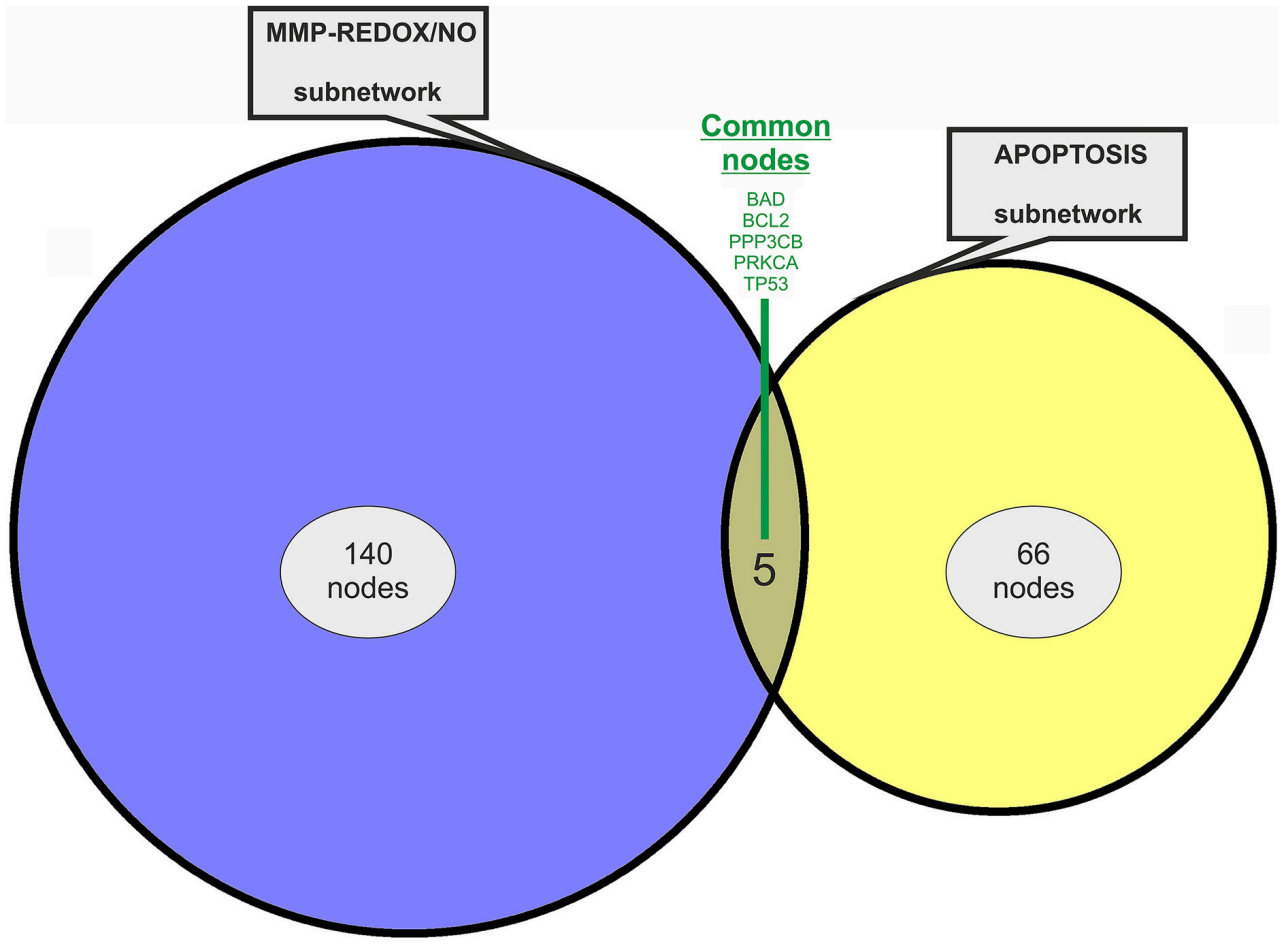
**Venn diagram showing information integration from the studied subnetworks**. The “MRN” (MMP-REDOX/NO) and “APOP” (apoptosis) network models contain five common nodes (genes/proteins).

**Figure 3:**
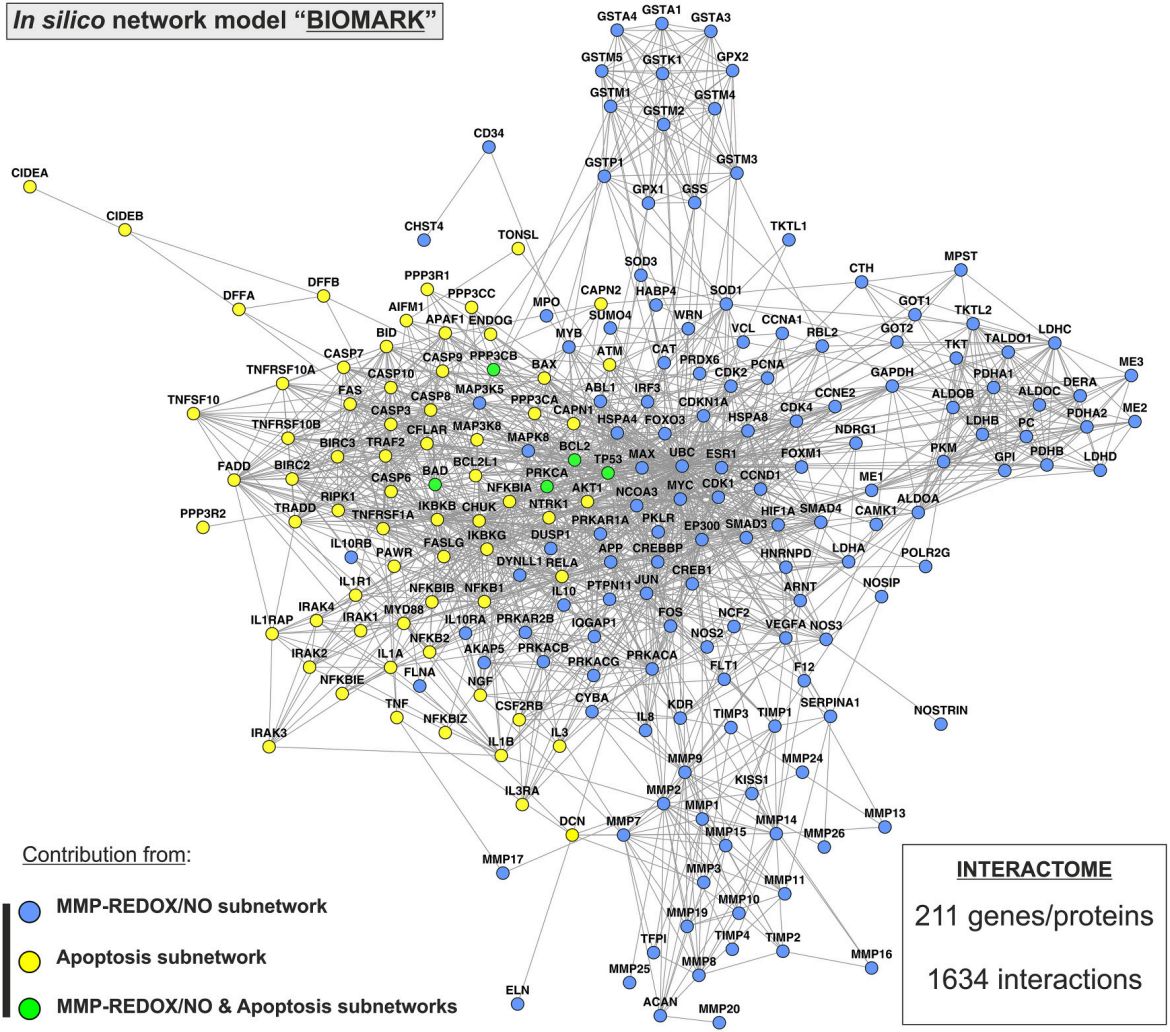
**“BIOMARK” interactome**. The network model of interactions between genes/protein belonging to “MRN” (MMP-REDOX/NO) and “APOP” (apoptosis) subnetworks or “BIOMARK” interactome was developed by using the STRING 10 database resource search tool, under a confidence score of 0.400, and by using “Databases” and “Experiments” as input options and visualized by plotting it with Cytoscape software. The subnetwork contribution of each gene/protein within the network is represented in the figure with blue-colored (MMP-REDOX/NO), yellow-colored (apoptosis), or green-colored nodes (contributing to both MMP-REDOX/NO and apoptosis subnetworks).

Two previously published subnetworks of gene/protein interactions characterizing the molecular landscape of MMP-REDOX/NO (“MRN”) and apoptosis (“APOP”) pathways were selected (Figure 1). The Venn diagram, which visualizes the level of subnetwork information integration, shows that both subnetworks contain five common nodes (genes/proteins) allowing communication with one another for constructing the “BIOMARK” interactome (Figure 2). In the case of non-existing direct crosstalk through common subnetwork nodes, it is possible to find common neighboring nodes (genes/proteins) to members of both subnetworks by the use of mathematical algorithms in the R environment. By using the STRING 10 (“Experiments” and “Databases”; confidence score of 0.400) and plotting with the Cytoscape software, we then developed the *in silico* network model “BIOMARK” (Figure 3) composed of 211 genes/proteins interconnecting through 1634 interactions.

### Identification of three key hub genes/proteins within the “BIOMARK” interactome suggests UBC, JUN, and MMP14 as putative host-derived biomarkers of periodontitis

**Figure 4:**
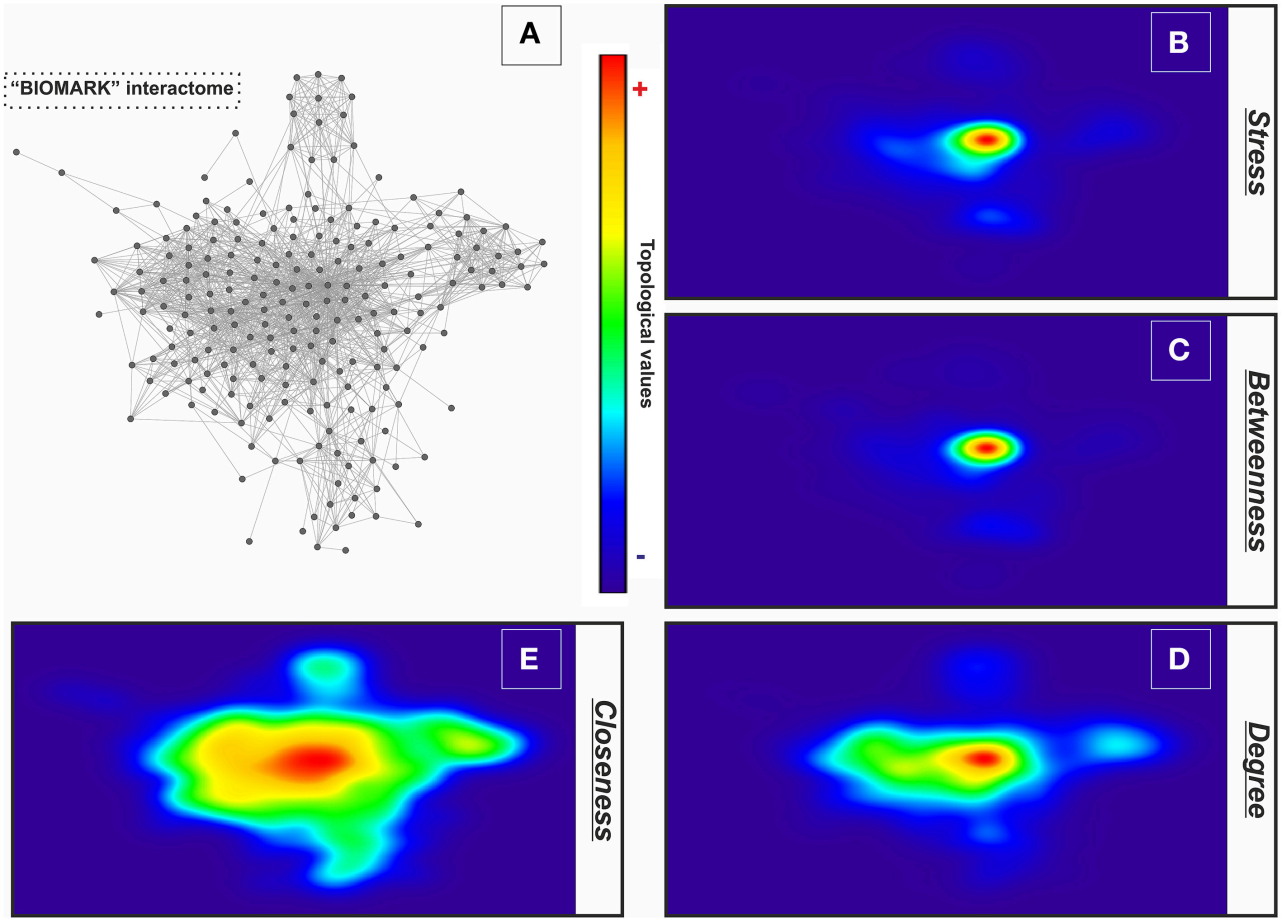
**GALANT plots of centrality values over the “BIOMARK” interactome**. Analysis of the topological network properties by Cytoscape and its 2D projection by the GALANT software over the “BIOMARK” interactome **(A)** show the areas of maximum values (red color) of stress **(B)**, betweenness **(C)**, degree **(D)**, and closeness centralities **(E)** of each node in this color-grading representation.

**Figure 5:**
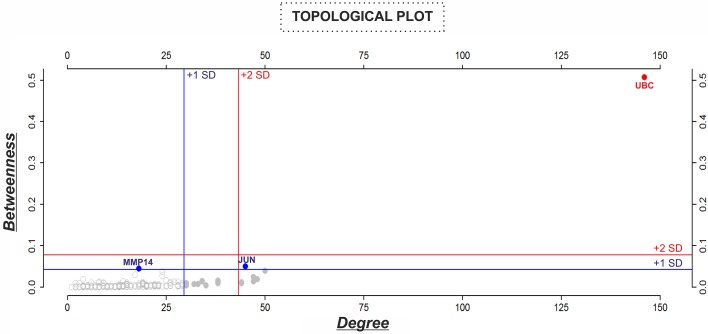
**Analysis of the topological properties (***betweenness*** vs. ***degree***) of genes/proteins belonging to the “BIOMARK” interactome**. Blue and red lines indicate the threshold value for each property or value/s above one (+1 SD, blue color) or two (+2 SD, red color) standard deviations of the mean. In the graphs, genes/proteins are represented by circles and plotted in blue or red when value/s fall within +1 or +2 SD, respectively. Values of centralities above +1 SD of the mean were selected to identify potential candidate host-derived biomarker/s.

For describing the global characteristics of the newly-developed interactome, we elucidated the topological network properties or centrality values (Supplementary Table 2). The measurements uncover the most central nodes (genes/proteins) of our model, representing “vulnerable” points within the “BIOMARK” interactome. Central genes/proteins can be thus considered putative biomarkers, because variations in these members can trigger more intense changes in the rest of the interactome than those in non- or less central members. After calculating each centrality value for the 211 “BIOMARK” gene/protein members and visualizing the data as a functional landscape projected onto the interactome, we identified network areas with high centrality values and, more specifically, Ubiquitin C (UBC) and Jun proto-oncogene (JUN) as hub-bottlenecks (HBs) of our model (Figure 4 and Table 1). These genes/proteins also displayed the highest values of *stress* and *closeness* centralities. Moreover, only a third gene/protein, MMP14 (an activator of host gelatinases), was identified as non-hub-bottleneck (NH-B) with a *betweenness* value over the threshold (Figure 5 and Table 1).

**Table 1:** **Nodes (genes/proteins) with the highest ***betweenness*** values from the “BIOMARK” interactome that integrates MMP-REDOX/NO (“MRN”) and apoptosis (“APOP”) subnetworks**.

**NAME/SYMBOL**	**—/HB/NH-B**	***Closeness***	***Stress***	***Degree***	***Betweenness***
*UBC*	HB	0.74204947	111,970	146	0.50695465
*JUN*	HB	0.53571429	17,440	45	0.04948189
*MMP14*	NH-B	0.47727273	10,116	18	0.04427346
*TP53*	—	0.53164557	12,626	50	0.03929094
*MMP2*	—	0.42510121	10,940	24	0.03761779
*MYB*	—	0.47191011	5590	17	0.02991401
*MMP9*	—	0.41666667	10,146	24	0.02747875
*RELA*	—	0.52369077	10,756	47	0.02417225
*EP300*	—	0.52631579	8960	48	0.01898303
*DFFB*	—	0.43659044	2568	4	0.01857965
*APP*	—	0.48498845	4704	25	0.01581576
*NFKB1*	—	0.5060241	7870	38	0.01551606
*CASP3*	—	0.48387097	5000	34	0.01418542
*TIMP1*	—	0.47511312	3998	14	0.01402902
*CHUK*	—	0.50724638	7092	47	0.01394735
*AKT1*	—	0.49065421	5388	34	0.01363533
*DCN*	—	0.37366548	2994	8	0.01291272
*MAP3K5*	—	0.47619048	3706	27	0.01285955
*TRAF2*	—	0.48498845	4716	28	0.01225608
*IKBKB*	—	0.5060241	5976	44	0.01139259
Average		0.4278415	2297.54	15.583	0.006697052
+1 SD		0.492552	10277.8	29.4	0.042061202
+2 SD		0.5572626	18,258	43.217	0.077425351

*Closeness, stress, and degree values are also shown. Centralities over the thresholds with value/s above one (+1 SD) or two (+2 SD) standard deviations of the mean are color-marked. Nodes identified as non-hub-bottlenecks and hub-bottlenecks (based on degree and betweenness centralities), with values above +1 SD of the mean, are represented as NH-B and HB, respectively*.

## Discussion

In the present study, our principal aim was to test the feasibility of creating an *in silico* model able to provide a molecular landscape of putative salivary biomarkers of periodontitis that could be later analyzed by wet laboratory techniques from patient samples. This model should be able to integrate a maximum number of genes/proteins from biological processes that are typically altered in periodontal inflammation (e.g., oxidative stress and apoptosis) into one single interactome.

According to our results, UBC, JUN, and MMP14 control the flow of biological information within the interactome integrating the MMP-REDOX/NO and apoptosis pathways. Their up- or down-regulation would critically affect the entire network, since they are the most central proteins of the “BIOMARK” interactome in comparison to the rest of network members. In a similar manner, if UBC, JUN, or MMP14 is disrupted by drug interaction, the entire network would be destroyed into small components. Thus, they represent three optimal therapeutic targets *in silico*, to be tested *in vitro* and/or *in vivo*. By applying the same criteria, the cumulative use of JUN, UBC, and MMP14 together with oral pathogenic bacteria-derived proteins (Gursoy et al., 2011) could be used as representative biomarkers of susceptibility to periodontitis for early intervention, if these central members are detectable in saliva from diseased individuals at the RNA or protein level (Kousvelari et al., 1990; Fábián et al., 2008; Cuevas-Córdoba and Santiago-García, 2014; Rall et al., 2015). UBC is a highly connected protein in the whole human protein-protein interaction network and it is involved in pathogenesis of different diseases (e.g., neurodegenerative diseases; Ullrich et al., 2010). Its use for the diagnosis of periodontitis may lack sensitivity and this could be considered as a potential limitation.

As a general conceptual framework, protein-protein interactions offer the opportunity to analyze the functional relationships among biological molecules (Gursoy et al., 2008). The more processes are integrated in the form of subnetworks in the interactome, the more accurate will our *in silico* model be to reflect the molecular pathogenesis of periodontal inflammation. An alternative possibility is, however, that other markers could also act as central members over the threshold upon the integration of additional subnetworks in the model, representing deregulated biological processes in periodontitis (e.g., epithelial cell adhesion; Haapasalmi et al., 1995; Gürsoy et al., 2015). These newly developed subnetworks might also be considered in the future as part of a systems biology approach utilized in different fields of medicine such as cancer, neurodegenerative, and psychiatric diseases (Rosado et al., 2011; Santana-Codina et al., 2013; ElRakaiby et al., 2014; Podder and Latha, 2014; Zeidán-Chuliá et al., 2014b; Ebhardt et al., 2015).

Saliva has a major importance in the maintenance of oral health, and, during the past two decades, it has been considered a potential specimen to detect oral and systemic diseases (Ji and Choi, 2015). Specific biomarkers have been identified from saliva, reflecting the three key features of pathogenic processes in periodontal disease, i.e., infection-induced inflammation, collagen degradation, and bone turnover (Zhang et al., 2009). Host- and bacteria-derived enzymes, proteins, and other inflammatory mediators appear to hold great promise as salivary biomarkers for the diagnosis of periodontal disease (Ramseier et al., 2009; Haigh et al., 2010; Salazar et al., 2013).

Saliva represents a non-invasive and safe study specimen, being especially useful in large-scale studies. During the onset and progress of periodontitis, inflammatory markers are released from cells present in the periodontium. Elevated levels of enzymes, cytokines, and biomarkers of connective tissue destruction and bone turnover can be found in saliva of periodontitis patients in comparison to their controls (Gursoy et al., 2009, 2010, 2013; Kinney et al., 2011; Lee et al., 2012). Furthermore, there is a considerable interest in applying sequencing and genotyping studies in various human populations. It has been demonstrated that saliva samples, in comparison to cheek swabs, provide a substantial increase in the amount of human DNA (Fábián et al., 2008). Although saliva may be used as a diagnostic tool for detecting periodontitis, there is only a subtle consensus on few salivary molecules to be used as putative markers of periodontitis (Gursoy et al., 2011; Salminen et al., 2014). The search for a novel biomarker is a costly and long-term process. With the aid of systems biology, however, it is possible to simultaneously analyze multiple candidate biomarkers within a network of interactions representing different biological processes (e.g., apoptosis, oxidative stress, and MMP secretion), which are characteristic of a given disease, such as periodontitis.

Up to our knowledge, our study is the first one combining MMP-REDOX/NO and apoptosis pathways that are well-known pathways in inflammatory diseases, including periodontitis, in an attempt to find regulative biomarkers. These two pathways that represent diminished tissue regeneration were taken as an example, since periodontitis is an outcome of a disrupted balance between tissue degeneration and regeneration. The selected pathways, however, form only a part of the whole cascade of events, and applying other additional models may give different results. Therefore, testing different underlying mechanisms in a combined manner would be beneficial to design putative biomarker groups for periodontitis.

In general, pathogenic pathways involved in the imbalance of connective tissue homeostasis in periodontitis are complex. Regarding our proposed candidate biomarkers, polyubiquitin-C is known as a precursor protein encoded by the *UBC* gene that is cleaved into the active ubiquitin monomer (Hanna et al., 2007). Among other processes, the ubiquitin conjugation system regulates protein degradation and signal transduction, playing a critical role in the regulation of innate and adaptive immunological responses (Liu et al., 2005). It seems that a decreased ubiquitin level can reduce the activation threshold of cells to environmental stressors, and UBC has been thus proposed as one promising candidate biomarker together with calmodulin-like protein 5 for the identification of newborns predisposed to develop atopic eczema (Holm et al., 2014). The Jun proto-oncogene (*JUN*) is a component of the AP-1 transcription factor, which is activated by several extracellular stimuli, such as proinflammatory cytokines and UV radiation, and plays a role in mediating the cellular response (Wisdom et al., 1999). For instance, c-Jun confers protection against UV-induced cellular apoptosis (Wisdom et al., 1999). It was recently shown that several genes involved in cellular apoptosis are deregulated in diseased gingival samples from periodontitis patients (Zeidán-Chuliá et al., 2014a). In fibroblasts, c-Jun has been suggested to activate cell death by acting as a transcriptional regulator (Bossy-Wetzel et al., 1997), likely having a similar role in periodontal inflammation. Finally, it is well-known that MMP14 is able to degrade collagen and gelatin, activate other MMPs relevant in periodontal inflammation progression, such as MMP2 and MMP13, shed cell surface proteins, and prevent collagen-induced apoptosis (Maquoi et al., 2012; Albrechtsen et al., 2013; Zeidán-Chuliá et al., 2013). To the best of our knowledge, there are no previous data reporting UBC and JUN as putative biomarkers of periodontitis. Our study group has previously analyzed salivary MMP14 concentrations as a potential biomarker for advanced periodontitis but failed to show any significant difference between periodontitis and non-periodontitis/gingivitis subjects (Gursoy et al., 2010). Instead, as an activator of main collagen degrading enzymes of gingiva like MMP2 and MMP9, which are known to be up-regulated in periodontitis (Zeidán-Chuliá et al., 2013; Haage et al., 2014), MMP14 may be a potential marker of inflammatory processes at early phases of periodontal pathogenesis.

On the basis of our findings, we propose the cumulative use of UBC, JUN, and MMP14, possibly in combination with oral pathogenic bacteria-derived proteins, as putative biomarkers for early detection of periodontitis to be tested either at the RNA or protein level in salivary samples. For example, high-throughput omics data found in other publicly available databases (e.g., Gene Expression Omnibus, Reactome, or BioGRID) could be used to validate our candidate biomarkers or further expand the BIOMARK interactome. Besides, the present systems biology-based approach may be used as an objective tool to identify, with measurable parameters, candidate molecular targets to treat this disease.

## Author contributions

FZ and UG conceived of the study; FZ, UK, EK, VO, and MG participated in its design and coordination; FZ and BN performed the *in silico* analyses; FZ and UG wrote the manuscript; EK, VO, and MG provided the critical revision of the manuscript; all authors read and approved the final manuscript.

### Conflict of interest statement

The authors declare that the research was conducted in the absence of any commercial or financial relationships that could be construed as a potential conflict of interest.
