# Supplementary material for: A Systems Biology Approach to Reveal Putative Host-Derived Biomarkers of Periodontitis by Network Topology Characterization of MMP-REDOX/NO and Apoptosis Integrated Pathways
Source: Front Cell Infect Microbiol. 2016 Jan 11;5:102. doi: 10.3389/fcimb.2015.00102 (PMC4707239; doi:10.3389/fcimb.2015.00102)
Supplement: Supplementary file 1 [file Table1.DOCX]

**SUPPLEMENTARY TABLE 1.** Genes/proteins in the “BIOMARK” interactome.

| **GENES/PROTEINS** | | |
| --- | --- | --- |
| **Gene symbol** | **Ensembl ID** (ENSP) | **Alias and/or description** |
| *ABL1* | ENSP00000361423 | c-abl oncogene 1, non-receptor tyrosine kinase |
| *ACAN* | ENSP00000387356 | Aggrecan |
| *AIFM1* | ENSP00000287295 | Apoptosis-inducing factor, mitochondrion-associated, 1 |
| *AKAP5* | ENSP00000315615 | A kinase (PRKA) anchor protein 5 |
| *AKT1* | ENSP00000270202 | v-akt murine thymoma viral oncogene homolog 1 |
| *ALDOA* | ENSP00000336927 | Aldolase A, fructose-bisphosphate |
| *ALDOB* | ENSP00000363988 | Aldolase B, fructose-bisphosphate |
| *ALDOC* | ENSP00000226253 | Aldolase C |
| *APAF1* | ENSP00000448165 | Apoptotic peptidase activating factor 1 |
| *APP* | ENSP00000284981 | Amyloid beta (A4) precursor protein |
| *ARNT* | ENSP00000351407 | Aryl hydrocarbon receptor nuclear translocator |
| *ATM* | ENSP00000278616 | Ataxia telangiectasia mutated |
| *BAD* | ENSP00000309103 | BCL2-associated agonist of cell death |
| *BAX* | ENSP00000293288 | BCL2-associated X protein |
| *BCL2* | ENSP00000329623 | B-cell CLL/lymphoma 2 |
| *BCL2L1* | ENSP00000302564 | BCL2-like 1 |
| *BID* | ENSP00000318822 | BH3 interacting domain death agonist |
| *BIRC2* | ENSP00000227758 | Baculoviral IAP repeat containing 2 |
| *BIRC3* | ENSP00000263464 | Baculoviral IAP repeat containing 3 |
| *CAMK1* | ENSP00000256460 | Calcium/calmodulin-dependent protein kinase I |
| *CAPN1* | ENSP00000279247 | Calpain 1, (mu/I) large subunit |
| *CAPN2* | ENSP00000295006 | Calpain 2, (m/II) large subunit |
| *CASP10* | ENSP00000286186 | Caspase 10, apoptosis-related cysteine peptidase |
| *CASP3* | ENSP00000311032 | Caspase 3, apoptosis-related cysteine peptidase |
| *CASP6* | ENSP00000265164 | Caspase 6, apoptosis-related cysteine peptidase |
| *CASP7* | ENSP00000358327 | Caspase 7, apoptosis-related cysteine peptidase |
| *CASP8* | ENSP00000351273 | Caspase 8, apoptosis-related cysteine peptidase |
| *CASP9* | ENSP00000330237 | Caspase 9, apoptosis-related cysteine peptidase |
| *CAT* | ENSP00000241052 | Catalase |
| *CCNA1* | ENSP00000255465 | Cyclin A1 |
| *CCND1* | ENSP00000227507 | Cyclin D1 |
| *CCNE2* | ENSP00000309181 | Cyclin E2 |
| *CD34* | ENSP00000310036 | CD34 molecule |
| *CDK1* | ENSP00000378699 | Cyclin-dependent kinase 1 |
| *CDK2* | ENSP00000266970 | Cyclin-dependent kinase 2 |
| *CDK4* | ENSP00000257904 | Cyclin-dependent kinase 4 |
| *CDKN1A* | ENSP00000244741 | Cyclin-dependent kinase inhibitor 1A (p21, Cip1) |
| *CFLAR* | ENSP00000312455 | CASP8 and FADD-like apoptosis regulator |
| *CHST4* | ENSP00000341206 | Carbohydrate (N-acetylglucosamine 6-O) sulfotransferase 4 |
| *CHUK* | ENSP00000359424 | Conserved helix-loop-helix ubiquitous kinase |
| *CIDEA* | ENSP00000320209 | Cell death-inducing DFFA-like effector a |
| *CIDEB* | ENSP00000258807 | Cell death-inducing DFFA-like effector b |
| *CREB1* | ENSP00000387699 | cAMP responsive element binding protein 1 |
| *CREBBP* | ENSP00000262367 | CREB binding protein |
| *CSF2RB* | ENSP00000384053 | Colony stimulating factor 2 receptor, beta, low-affinity (granulocyte-macrophage) |
| *CTH* | ENSP00000359976 | Cystathionase (cystathionine gamma-lyase) |
| *CYBA* | ENSP00000261623 | Cytochrome b-245, alpha polypeptide |
| *DCN* | ENSP00000052754 | Decorin |
| *DERA* | ENSP00000416583 | Deoxyribose-phosphate aldolase |
| *DFFA* | ENSP00000366237 | DNA fragmentation factor, 45kDa, alpha polypeptide |
| *DFFB* | ENSP00000367454 | DNA fragmentation factor, 40kDa, beta polypeptide |
| *DUSP1* | ENSP00000239223 | Dual specificity phosphatase 1 |
| *DYNLL1* | ENSP00000242577 | Dynein, light chain, LC8-type 1 |
| *ELN* | ENSP00000252034 | Elastin |
| *ENDOG* | ENSP00000361725 | Endonuclease G |
| *EP300* | ENSP00000263253 | E1A binding protein p300 |
| *ESR1* | ENSP00000206249 | Estrogen receptor 1 |
| *F12* | ENSP00000253496 | Coagulation factor XII (Hageman factor) |
| *FADD* | ENSP00000301838 | Fas (TNFRSF6)-associated via death domain |
| *FAS* | ENSP00000347979 | Fas (TNF receptor superfamily, member 6) |
| *FASLG* | ENSP00000356694 | Fas ligand (TNF superfamily, member 6) |
| *FLNA* | ENSP00000358866 | Filamin A |
| *FLT1* | ENSP00000282397 | Fms-related tyrosine kinase 1 |
| *FOS* | ENSP00000306245 | FBJ murine osteosarcoma viral oncogene homolog |
| *FOXM1* | ENSP00000342307 | Forkhead box M1 |
| *FOXO3* | ENSP00000339527 | Forkhead box O3 |
| *GAPDH* | ENSP00000229239 | Glyceraldehyde-3-phosphate dehydrogenase |
| *GOT1* | ENSP00000359539 | Glutamic-oxaloacetic transaminase 1, soluble (aspartate aminotransferase 1) |
| *GOT2* | ENSP00000245206 | Glutamic-oxaloacetic transaminase 2, mitochondrial (aspartate aminotransferase 2) |
| *GPI* | ENSP00000405573 | Glucose-6-phosphate isomerase |
| *GPX1* | ENSP00000407375 | Glutathione peroxidase 1 |
| *GPX2* | ENSP00000374265 | Glutathione peroxidase 2 |
| *GSS* | ENSP00000216951 | Glutathione synthetase |
| *GSTA1* | ENSP00000335620 | Glutathione S-transferase alpha 1 |
| *GSTA3* | ENSP00000211122 | Glutathione S-transferase alpha 3 |
| *GSTA4* | ENSP00000359998 | Glutathione S-transferase alpha 4 |
| *GSTK1* | ENSP00000431049 | Glutathione S-transferase kappa 1 |
| *GSTM1* | ENSP00000311469 | Glutathione S-transferase mu 1 |
| *GSTM2* | ENSP00000241337 | Glutathione S-transferase mu 2 |
| *GSTM3* | ENSP00000256594 | Glutathione S-transferase mu 3 |
| *GSTM4* | ENSP00000358851 | Glutathione S-transferase mu 4 |
| *GSTM5* | ENSP00000256593 | Glutathione S-transferase mu 5 |
| *GSTP1* | ENSP00000381607 | Glutathione S-transferase pi 1 |
| *HABP4* | ENSP00000364398 | Hyaluronan binding protein 4 |
| *HIF1A* | ENSP00000338018 | Hypoxia inducible factor 1, alpha subunit (basic helix-loop-helix transcription factor) |
| *HNRNPD* | ENSP00000313199 | Heterogeneous nuclear ribonucleoprotein D |
| *HSPA4* | ENSP00000302961 | Heat shock 70kDa protein 4 |
| *HSPA8* | ENSP00000227378 | Heat shock 70kDa protein 8 |
| *IKBKB* | ENSP00000430684 | Inhibitor of kappa light polypeptide gene enhancer in B-cells, kinase beta |
| *IKBKG* | ENSP00000358622 | Inhibitor of kappa light polypeptide gene enhancer in B-cells, kinase gamma |
| *IL10* | ENSP00000412237 | Interleukin 10 |
| *IL10RA* | ENSP00000227752 | Interleukin 10 receptor, alpha |
| *IL10RB* | ENSP00000290200 | Interleukin 10 receptor, beta |
| *IL1A* | ENSP00000263339 | Interleukin 1, alpha |
| *IL1B* | ENSP00000263341 | Interleukin 1, beta |
| *IL1R1* | ENSP00000233946 | Interleukin 1 receptor, type I |
| *IL1RAP* | ENSP00000314807 | Interleukin 1 receptor accessory protein |
| *IL3* | ENSP00000296870 | Interleukin 3 |
| *IL3RA* | ENSP00000327890 | Interleukin 3 receptor, alpha |
| *IL8* | ENSP00000306512 | Interleukin 8 |
| *IQGAP1* | ENSP00000268182 | IQ motif containing GTPase activating protein 1 |
| *IRAK1* | ENSP00000358997 | Interleukin-1 receptor-associated kinase 1 |
| *IRAK2* | ENSP00000256458 | Interleukin-1 receptor-associated kinase 2 |
| *IRAK3* | ENSP00000261233 | Interleukin-1 receptor-associated kinase 3 |
| *IRAK4* | ENSP00000390651 | Interleukin-1 receptor-associated kinase 4 |
| *IRF3* | ENSP00000310127 | Interferon regulatory factor 3 |
| *JUN* | ENSP00000360266 | Jun proto-oncogene |
| *KDR* | ENSP00000263923 | Kinase insert domain receptor (a type III receptor tyrosine kinase) |
| *KISS1* | ENSP00000356162 | KiSS-1 metastasis-suppressor |
| *LDHA* | ENSP00000445175 | Lactate dehydrogenase A |
| *LDHB* | ENSP00000229319 | Lactate dehydrogenase B |
| *LDHC* | ENSP00000280704 | Lactate dehydrogenase C |
| *LDHD* | ENSP00000300051 | Lactate dehydrogenase D |
| *MAP3K5* | ENSP00000351908 | Mitogen-activated protein kinase kinase kinase 5 |
| *MAPK8* | ENSP00000353483 | Mitogen-activated protein kinase 8 |
| *MAX* | ENSP00000351490 | MYC associated factor X |
| *ME1* | ENSP00000358719 | Malic enzyme 1, NADP(+)-dependent, cytosolic |
| *ME2* | ENSP00000321070 | Malic enzyme 2, NAD(+)-dependent, mitochondrial |
| *ME3* | ENSP00000352657 | Malic enzyme 3, NADP(+)-dependent, mitochondrial |
| *MMP1* | ENSP00000322788 | Matrix metallopeptidase 1 |
| *MMP10* | ENSP00000279441 | Matrix metallopeptidase 10 |
| *MMP11* | ENSP00000215743 | Matrix metallopeptidase 11 |
| *MMP13* | ENSP00000260302 | Matrix metallopeptidase 13 |
| *MMP14* | ENSP00000308208 | Matrix metallopeptidase 14 |
| *MMP15* | ENSP00000219271 | Matrix metallopeptidase 15 |
| *MMP16* | ENSP00000286614 | Matrix metallopeptidase 16 |
| *MMP17* | ENSP00000353767 | Matrix metallopeptidase 17 |
| *MMP19* | ENSP00000313437 | Matrix metallopeptidase 19 |
| *MMP2* | ENSP00000219070 | Matrix metallopeptidase 2 |
| *MMP20* | ENSP00000260228 | Matrix metallopeptidase 20 |
| *MMP24* | ENSP00000246186 | Matrix metallopeptidase 24 |
| *MMP25* | ENSP00000337816 | Matrix metallopeptidase 25 |
| *MMP26* | ENSP00000300762 | Matrix metallopeptidase 26 |
| *MMP3* | ENSP00000299855 | Matrix metallopeptidase 3 |
| *MMP7* | ENSP00000260227 | Matrix metallopeptidase 7 |
| *MMP8* | ENSP00000236826 | Matrix metallopeptidase 8 |
| *MMP9* | ENSP00000361405 | Matrix metallopeptidase 9 |
| *MPO* | ENSP00000225275 | Myeloperoxidase |
| *MPST* | ENSP00000380318 | Mercaptopyruvate sulfurtransferase |
| *MYB* | ENSP00000339992 | v-myb myeloblastosis viral oncogene homolog |
| *MYC* | ENSP00000367207 | v-myc myelocytomatosis viral oncogene homolog |
| *MYD88* | ENSP00000401399 | Myeloid differentiation primary response 88 |
| *NCF2* | ENSP00000356505 | Neutrophil cytosolic factor 2 |
| *NCOA3* | ENSP00000361066 | Nuclear receptor coactivator 3 |
| *NDRG1* | ENSP00000319977 | N-myc downstream regulated 1 |
| *NFKB1* | ENSP00000226574 | Nuclear factor of kappa light polypeptide gene enhancer in B-cells 1 |
| *NFKB2* | ENSP00000358983 | Nuclear factor of kappa light polypeptide gene enhancer in B-cells 2 (p49/p100) |
| *NFKBIA* | ENSP00000216797 | Nuclear factor of kappa light polypeptide gene enhancer in B-cells inhibitor, alpha |
| *NFKBIB* | ENSP00000312988 | Nuclear factor of kappa light polypeptide gene enhancer in B-cells inhibitor, beta |
| *NFKBIE* | ENSP00000275015 | Nuclear factor of kappa light polypeptide gene enhancer in B-cells inhibitor, epsilon |
| *NFKBIZ* | ENSP00000325663 | Nuclear factor of kappa light polypeptide gene enhancer in B-cells inhibitor, zeta |
| *NGF* | ENSP00000358525 | Nerve growth factor (beta polypeptide) |
| *NOS2* | ENSP00000327251 | Nitric oxide synthase 2, inducible |
| *NOS3* | ENSP00000297494 | Nitric oxide synthase 3 (endothelial cell) |
| *NOSIP* | ENSP00000375726 | Nitric oxide synthase interacting protein |
| *NOSTRIN* | ENSP00000394051 | Nitric oxide synthase trafficker |
| *NTRK1* | ENSP00000431418 | Neurotrophic tyrosine kinase, receptor, type 1 |
| *PAWR* | ENSP00000328088 | PRKC, apoptosis, WT1, regulator |
| *PC* | ENSP00000377527 | Pyruvate carboxylase |
| *PCNA* | ENSP00000368438 | Proliferating cell nuclear antigen |
| *PDHA1* | ENSP00000369134 | Pyruvate dehydrogenase (lipoamide) alpha 1 |
| *PDHA2* | ENSP00000295266 | Pyruvate dehydrogenase (lipoamide) alpha 2 |
| *PDHB* | ENSP00000307241 | Pyruvate dehydrogenase (lipoamide) beta |
| *PKLR* | ENSP00000339933 | Pyruvate kinase, liver and RBC |
| *PKM* | ENSP00000320171 | Pyruvate kinase |
| *POLR2G* | ENSP00000301788 | Polymerase (RNA) II (DNA directed) polypeptide G |
| *PPP3CA* | ENSP00000378323 | Protein phosphatase 3, catalytic subunit, alpha isozyme |
| *PPP3CB* | ENSP00000378306 | Protein phosphatase 3, catalytic subunit, beta isozyme |
| *PPP3CC* | ENSP00000240139 | Protein phosphatase 3, catalytic subunit, gamma isozyme |
| *PPP3R1* | ENSP00000234310 | Protein phosphatase 3, regulatory subunit B, alpha |
| *PPP3R2* | ENSP00000363939 | Protein phosphatase 3, regulatory subunit B, beta |
| *PRDX6* | ENSP00000342026 | Peroxiredoxin 6 |
| *PRKACA* | ENSP00000309591 | Protein kinase, cAMP-dependent, catalytic, alpha |
| *PRKACB* | ENSP00000359719 | Protein kinase, cAMP-dependent, catalytic, beta |
| *PRKACG* | ENSP00000366488 | Protein kinase, cAMP-dependent, catalytic, gamma |
| *PRKAR1A* | ENSP00000351410 | Protein kinase, cAMP-dependent, regulatory, type I, alpha |
| *PRKAR2B* | ENSP00000265717 | Protein kinase, cAMP-dependent, regulatory, type II, beta |
| *PRKCA* | ENSP00000408695 | Protein kinase C, alpha |
| *PTPN11* | ENSP00000340944 | Protein tyrosine phosphatase, non-receptor type 11 |
| *RBL2* | ENSP00000262133 | Retinoblastoma-like 2 (p130) |
| *RELA* | ENSP00000384273 | v-rel reticuloendotheliosis viral oncogene homolog A |
| *RIPK1* | ENSP00000259808 | Receptor (TNFRSF)-interacting serine-threonine kinase 1 |
| *SERPINA1* | ENSP00000348068 | Serpin peptidase inhibitor, clade A (alpha-1 antiproteinase, antitrypsin), member 1 |
| *SMAD3* | ENSP00000332973 | SMAD family member 3 |
| *SMAD4* | ENSP00000341551 | SMAD family member 4; |
| *SOD1* | ENSP00000270142 | Superoxide dismutase 1 |
| *SOD3* | ENSP00000371554 | Superoxide dismutase 3, extracellular |
| *SUMO4* | ENSP00000318635 | SMT3 suppressor of mif two 3 homolog 4 |
| *TALDO1* | ENSP00000321259 | Transaldolase 1 |
| *TFPI* | ENSP00000233156 | Tissue factor pathway inhibitor (lipoprotein-associated coagulation inhibitor) |
| *TIMP1* | ENSP00000218388 | TIMP metallopeptidase inhibitor 1 |
| *TIMP2* | ENSP00000262768 | TIMP metallopeptidase inhibitor 2 |
| *TIMP3* | ENSP00000266085 | TIMP metallopeptidase inhibitor 3 |
| *TIMP4* | ENSP00000287814 | TIMP metallopeptidase inhibitor 4 |
| *TKT* | ENSP00000405455 | Transketolase |
| *TKTL1* | ENSP00000358931 | Transketolase-like 1 |
| *TKTL2* | ENSP00000280605 | Transketolase-like 2 |
| *TNF* | ENSP00000398698 | Tumor necrosis factor |
| *TNFRSF10A* | ENSP00000221132 | Tumor necrosis factor receptor superfamily, member 10a |
| *TNFRSF10B* | ENSP00000276431 | Tumor necrosis factor receptor superfamily, member 10b |
| *TNFRSF1A* | ENSP00000162749 | Tumor necrosis factor receptor superfamily, member 1A |
| *TNFSF10* | ENSP00000241261 | Tumor necrosis factor (ligand) superfamily, member 10 |
| *TONSL* | ENSP00000386239 | Tonsoku-like, DNA repair protein |
| *TP53* | ENSP00000269305 | Tumor protein p53 |
| *TRADD* | ENSP00000341268 | TNFRSF1A-associated via death domain |
| *TRAF1* | ENSP00000362994 | TNF receptor-associated factor 1 |
| *TRAF2* | ENSP00000247668 | TNF receptor-associated factor 2 |
| *UBC* | ENSP00000344818 | Ubiquitin C |
| *VCL* | ENSP00000211998 | Vinculin |
| *VEGFA* | ENSP00000361125 | Vascular endothelial growth factor A |
| *WRN* | ENSP00000298139 | Werner syndrome, RecQ helicase-like |
