# Supplementary material for: A Systems Biology Approach to Reveal Putative Host-Derived Biomarkers of Periodontitis by Network Topology Characterization of MMP-REDOX/NO and Apoptosis Integrated Pathways
Source: Front Cell Infect Microbiol. 2016 Jan 11;5:102. doi: 10.3389/fcimb.2015.00102 (PMC4707239; doi:10.3389/fcimb.2015.00102)
Supplement: Supplementary file 2 [file Table2.docx]

**SUPPLEMENTARY TABLE 2.** Centrality values (*closeness*, *stress*, *degree*, and *betweenness*) for each node (gene/protein) belonging to the “BIOMARK” interactome that integrates MMP-REDOX/NO (“MRN”) and apoptosis (“APOP”) subnetworks. Centralities over the thresholds with value/s above one (+ 1 SD) or two (+ 2 SD) standard deviations of the mean are color-marked. Nodes identified as non-hub-bottlenecks and hub-bottlenecks (based on *degree* and *betweenness* centralities), with values above + 1 SD of the mean, are represented as NH-B and HB, respectively. Table sorted by *betweenness* values.

| **NAME / SYMBOL** | **--- / HB / NH-B** | ***Closeness*** | ***Stress*** | ***Degree*** | ***Betweenness*** |
| --- | --- | --- | --- | --- | --- |
| ***UBC*** | HB | 0.74204947 | 111970 | 146 | 0.50695465 |
| ***JUN*** | HB | 0.53571429 | 17440 | 45 | 0.04948189 |
| ***MMP14*** | NH-B | 0.47727273 | 10116 | 18 | 0.04427346 |
| *TP53* | --- | 0.53164557 | 12626 | 50 | 0.03929094 |
| *MMP2* | **---** | 0.42510121 | 10940 | 24 | 0.03761779 |
| *MYB* | **---** | 0.47191011 | 5590 | 17 | 0.02991401 |
| *MMP9* | **---** | 0.41666667 | 10146 | 24 | 0.02747875 |
| *RELA* | --- | 0.52369077 | 10756 | 47 | 0.02417225 |
| *EP300* | --- | 0.52631579 | 8960 | 48 | 0.01898303 |
| *DFFB* | **---** | 0.43659044 | 2568 | 4 | 0.01857965 |
| *APP* | **---** | 0.48498845 | 4704 | 25 | 0.01581576 |
| *NFKB1* | --- | 0.5060241 | 7870 | 38 | 0.01551606 |
| *CASP3* | --- | 0.48387097 | 5000 | 34 | 0.01418542 |
| *TIMP1* | **---** | 0.47511312 | 3998 | 14 | 0.01402902 |
| *CHUK* | --- | 0.50724638 | 7092 | 47 | 0.01394735 |
| *AKT1* | --- | 0.49065421 | 5388 | 34 | 0.01363533 |
| *DCN* | **---** | 0.37366548 | 2994 | 8 | 0.01291272 |
| *MAP3K5* | **---** | 0.47619048 | 3706 | 27 | 0.01285955 |
| *TRAF2* | **---** | 0.48498845 | 4716 | 28 | 0.01225608 |
| *IKBKB* | --- | 0.5060241 | 5976 | 44 | 0.01139259 |
| *BAD* | **---** | 0.39848197 | 7332 | 19 | 0.01130317 |
| *PKM* | **---** | 0.46153846 | 5776 | 26 | 0.01111483 |
| *BCL2* | --- | 0.49528302 | 4622 | 38 | 0.01100771 |
| *NOS3* | **---** | 0.46153846 | 1954 | 15 | 0.0106552 |
| *KISS1* | **---** | 0.35836177 | 2760 | 6 | 0.01055459 |
| *SERPINA1* | **---** | 0.45454545 | 2134 | 8 | 0.01053265 |
| *ACAN* | **---** | 0.34883721 | 1976 | 9 | 0.00996687 |
| *PRKACA* | **---** | 0.48165138 | 5688 | 30 | 0.00962956 |
| *CD34* | **---** | 0.32208589 | 1626 | 2 | 0.00952381 |
| *CIDEB* | **---** | 0.30656934 | 1160 | 3 | 0.00952381 |
| *SOD1* | **---** | 0.4595186 | 4020 | 19 | 0.00945224 |
| *CREBBP* | **---** | 0.50724638 | 4648 | 44 | 0.00943603 |
| *FOS* | **---** | 0.4964539 | 5352 | 26 | 0.00942914 |
| *MYC* | **---** | 0.50970874 | 4208 | 38 | 0.00933251 |
| *MMP15* | **---** | 0.46153846 | 3640 | 9 | 0.00883853 |
| *PRKCA* | **---** | 0.47085202 | 3502 | 19 | 0.00845275 |
| *GSTP1* | **---** | 0.45851528 | 4504 | 18 | 0.00841924 |
| *TNFRSF1A* | **---** | 0.48387097 | 2882 | 29 | 0.00791068 |
| *MMP1* | --- | 0.39473684 | 4014 | 15 | 0.00759848 |
| *MAPK8* | **---** | 0.49411765 | 3614 | 33 | 0.00758464 |
| *MMP7* | **---** | 0.375 | 2578 | 15 | 0.0073616 |
| *NFKBIA* | **---** | 0.49065421 | 2732 | 32 | 0.00698688 |
| *ALDOA* | **---** | 0.46563193 | 3012 | 22 | 0.00688083 |
| *VEGFA* | **---** | 0.48387097 | 3066 | 19 | 0.00680647 |
| *ME1* | **---** | 0.4595186 | 3988 | 13 | 0.00661869 |
| *GSTM1* | **---** | 0.44871795 | 3346 | 15 | 0.00583223 |
| *LDHB* | **---** | 0.45258621 | 3054 | 23 | 0.0058235 |
| *NGF* | **---** | 0.46979866 | 2648 | 15 | 0.00570292 |
| *PRKACB* | **---** | 0.46563193 | 4348 | 22 | 0.00544916 |
| *PDHA1* | **---** | 0.4496788 | 3270 | 20 | 0.00538729 |
| *FASLG* | **---** | 0.47619048 | 2438 | 21 | 0.0052394 |
| *IL1B* | **---** | 0.40856031 | 1714 | 16 | 0.00503603 |
| *PTPN11* | **---** | 0.46770601 | 2120 | 18 | 0.00502655 |
| *GSS* | **---** | 0.44680851 | 3396 | 16 | 0.00499842 |
| *HIF1A* | **---** | 0.48723898 | 1880 | 29 | 0.00475749 |
| *GSTM3* | **---** | 0.44491525 | 3368 | 15 | 0.00468427 |
| *PDHB* | **---** | 0.44776119 | 3134 | 19 | 0.00456542 |
| *MYD88* | **---** | 0.46666667 | 2812 | 23 | 0.00435867 |
| *PC* | --- | 0.44210526 | 2928 | 13 | 0.00433075 |
| *ARNT* | **---** | 0.48387097 | 1588 | 22 | 0.00429852 |
| *CASP8* | **---** | 0.48951049 | 2392 | 35 | 0.00399754 |
| *IKBKG* | **---** | 0.47835991 | 2364 | 30 | 0.00398179 |
| *CASP7* | **---** | 0.45553145 | 1522 | 17 | 0.00394844 |
| *HSPA4* | **---** | 0.47404063 | 1382 | 26 | 0.00394182 |
| *GSTM2* | **---** | 0.44397463 | 2918 | 14 | 0.00389258 |
| *GAPDH* | **---** | 0.46875 | 1426 | 20 | 0.00386449 |
| *ESR1* | **---** | 0.48837209 | 2138 | 28 | 0.00380167 |
| *PRDX6* | **---** | 0.45258621 | 1210 | 9 | 0.00379936 |
| *F12* | **---** | 0.45064378 | 950 | 5 | 0.00374154 |
| *ABL1* | **---** | 0.47404063 | 1594 | 22 | 0.00349685 |
| *BID* | **---** | 0.47191011 | 1850 | 25 | 0.00334443 |
| *PPP3CA* | **---** | 0.46666667 | 1940 | 13 | 0.00331818 |
| *TIMP3* | **---** | 0.38321168 | 834 | 7 | 0.00325553 |
| *MAX* | **---** | 0.44117647 | 1306 | 20 | 0.00321967 |
| *GSTK1* | **---** | 0.44210526 | 2628 | 13 | 0.00321583 |
| *GSTM4* | **---** | 0.44210526 | 2628 | 13 | 0.00321583 |
| *GSTM5* | **---** | 0.44210526 | 2628 | 13 | 0.00321583 |
| *CDKN1A* | **---** | 0.4805492 | 1302 | 29 | 0.0030685 |
| *HSPA8* | **---** | 0.47404063 | 1000 | 21 | 0.00305781 |
| *GPX1* | **---** | 0.37906137 | 1324 | 14 | 0.00289308 |
| *IL10RA* | **---** | 0.43478261 | 964 | 5 | 0.00288769 |
| *FADD* | **---** | 0.47085202 | 1546 | 28 | 0.00287585 |
| *CDK1* | **---** | 0.47297297 | 1486 | 23 | 0.00282703 |
| *CYBA* | **---** | 0.45851528 | 1510 | 6 | 0.00275988 |
| *CSF2RB* | **---** | 0.4496788 | 1588 | 11 | 0.00266348 |
| *CREB1* | **---** | 0.47619048 | 1276 | 25 | 0.00262737 |
| *BIRC3* | **---** | 0.47511312 | 1020 | 26 | 0.00255132 |
| *BAX* | **---** | 0.46770601 | 1570 | 22 | 0.00252313 |
| *NOS2* | **---** | 0.45356371 | 874 | 10 | 0.00249292 |
| *IL1R1* | **---** | 0.45553145 | 1658 | 14 | 0.002492 |
| *TNF* | **---** | 0.34768212 | 808 | 4 | 0.00246932 |
| *PRKACG* | **---** | 0.40152964 | 1800 | 18 | 0.0023849 |
| *BCL2L1* | **---** | 0.46357616 | 1418 | 20 | 0.00236067 |
| *LDHC* | **---** | 0.35714286 | 630 | 22 | 0.00235512 |
| *KDR* | **---** | 0.4516129 | 730 | 11 | 0.00222503 |
| *IL10* | **---** | 0.37769784 | 642 | 8 | 0.0021883 |
| *SMAD3* | **---** | 0.47511312 | 1230 | 24 | 0.00216121 |
| *CCND1* | **---** | 0.47727273 | 942 | 24 | 0.00214667 |
| *RIPK1* | **---** | 0.47404063 | 1652 | 28 | 0.00209418 |
| *PRKAR1A* | **---** | 0.45454545 | 1794 | 16 | 0.00207395 |
| *CFLAR* | **---** | 0.47191011 | 1418 | 25 | 0.00206716 |
| *IRAK4* | **---** | 0.44776119 | 1492 | 13 | 0.00197411 |
| *CAT* | **---** | 0.44776119 | 656 | 9 | 0.00194652 |
| *CDK2* | **---** | 0.46875 | 910 | 22 | 0.00186729 |
| *DFFA* | **---** | 0.33227848 | 316 | 4 | 0.00183647 |
| *IL1RAP* | **---** | 0.44585987 | 1296 | 11 | 0.00181451 |
| *RBL2* | **---** | 0.44871795 | 982 | 13 | 0.0018145 |
| *IRAK2* | **---** | 0.44585987 | 1360 | 11 | 0.00178365 |
| *PRKAR2B* | **---** | 0.44871795 | 1378 | 14 | 0.00164772 |
| *CDK4* | **---** | 0.46153846 | 642 | 23 | 0.00157718 |
| *SMAD4* | **---** | 0.46357616 | 824 | 20 | 0.00152698 |
| *BIRC2* | **---** | 0.46770601 | 640 | 24 | 0.00152601 |
| *CASP10* | **---** | 0.41666667 | 784 | 25 | 0.00150789 |
| *TRADD* | **---** | 0.46875 | 1108 | 24 | 0.0014115 |
| *IQGAP1* | **---** | 0.44585987 | 832 | 10 | 0.00140583 |
| *TKT* | **---** | 0.44871795 | 1226 | 15 | 0.00135951 |
| *TKTL2* | **---** | 0.44871795 | 1226 | 15 | 0.00135951 |
| *IL1A* | **---** | 0.38461538 | 606 | 12 | 0.00129582 |
| *NFKB2* | **---** | 0.45652174 | 948 | 15 | 0.00125478 |
| *FOXO3* | **---** | 0.46770601 | 554 | 20 | 0.00121666 |
| *CAPN1* | **---** | 0.45258621 | 558 | 10 | 0.00110134 |
| *VCL* | **---** | 0.44210526 | 358 | 6 | 0.00109319 |
| *ALDOB* | **---** | 0.44585987 | 1012 | 16 | 0.00105648 |
| *CASP6* | **---** | 0.45751634 | 458 | 14 | 0.00105166 |
| *IRF3* | **---** | 0.46563193 | 632 | 13 | 0.00103838 |
| *LDHA* | **---** | 0.45751634 | 544 | 14 | 0.00103287 |
| *CASP9* | **---** | 0.46052632 | 540 | 15 | 0.00099816 |
| *IL3* | **---** | 0.37300178 | 464 | 9 | 0.00091476 |
| *IRAK1* | **---** | 0.39325843 | 478 | 10 | 0.00089833 |
| *NCF2* | **---** | 0.35472973 | 240 | 5 | 0.00086284 |
| *NCOA3* | **---** | 0.47085202 | 620 | 20 | 0.00081283 |
| *FAS* | **---** | 0.46770601 | 520 | 21 | 0.00071815 |
| *AIFM1* | **---** | 0.44025157 | 526 | 8 | 0.00069919 |
| *ALDOC* | **---** | 0.44491525 | 884 | 15 | 0.00068984 |
| *DERA* | **---** | 0.44491525 | 884 | 15 | 0.00068984 |
| *GPI* | **---** | 0.44491525 | 884 | 15 | 0.00068984 |
| *TALDO1* | **---** | 0.44491525 | 884 | 15 | 0.00068984 |
| *PCNA* | **---** | 0.45258621 | 200 | 15 | 0.00064817 |
| *MMP8* | **---** | 0.35058431 | 518 | 12 | 0.00062799 |
| *PPP3CB* | **---** | 0.4496788 | 672 | 7 | 0.00062348 |
| *GOT2* | **---** | 0.44025157 | 488 | 9 | 0.00061142 |
| *TNFRSF10B* | **---** | 0.46255507 | 680 | 19 | 0.00059748 |
| *APAF1* | **---** | 0.45652174 | 370 | 17 | 0.00058711 |
| *PDHA2* | **---** | 0.34596376 | 320 | 18 | 0.00051261 |
| *FLNA* | **---** | 0.35 | 176 | 3 | 0.00051016 |
| *TNFRSF10A* | **---** | 0.45652174 | 576 | 15 | 0.0004757 |
| *IL8* | **---** | 0.46460177 | 520 | 9 | 0.00045552 |
| *CTH* | **---** | 0.43659044 | 432 | 7 | 0.00045439 |
| *TIMP2* | **---** | 0.34313725 | 126 | 7 | 0.00044891 |
| *TRAF1* | **---** | 0.46255507 | 410 | 19 | 0.0004312 |
| *GOT1* | **---** | 0.43933054 | 424 | 8 | 0.00042494 |
| *ATM* | **---** | 0.45553145 | 176 | 13 | 0.00040112 |
| *GPX2* | **---** | 0.3343949 | 148 | 12 | 0.00037118 |
| *PPP3CC* | **---** | 0.44025157 | 500 | 7 | 0.00033359 |
| *PPP3R1* | **---** | 0.44025157 | 500 | 7 | 0.00033359 |
| *MMP3* | **---** | 0.3343949 | 84 | 4 | 0.00028591 |
| *HNRNPD* | **---** | 0.44491525 | 100 | 8 | 0.00028138 |
| *MPST* | **---** | 0.43568465 | 382 | 6 | 0.00026361 |
| *MMP17* | **---** | 0.30656934 | 46 | 2 | 0.00024452 |
| *IL3RA* | **---** | 0.33980583 | 100 | 7 | 0.0002366 |
| *DYNLL1* | **---** | 0.44303797 | 70 | 9 | 0.00021271 |
| *CCNA1* | **---** | 0.43933054 | 56 | 9 | 0.00020187 |
| *CAPN2* | **---** | 0.44025157 | 80 | 7 | 0.00019879 |
| *AKAP5* | **---** | 0.34596376 | 58 | 8 | 0.00018553 |
| *MMP11* | **---** | 0.34653465 | 68 | 7 | 0.00017083 |
| *CCNE2* | **---** | 0.4496788 | 142 | 11 | 0.00014997 |
| *MMP19* | **---** | 0.34941764 | 54 | 10 | 0.00013663 |
| *NTRK1* | **---** | 0.43841336 | 50 | 6 | 0.00012763 |
| *NFKBIB* | **---** | 0.39923954 | 88 | 11 | 0.00011652 |
| *MMP10* | **---** | 0.34825871 | 66 | 8 | 0.00011314 |
| *NDRG1* | **---** | 0.44303797 | 104 | 8 | 0.00010445 |
| *WRN* | **---** | 0.44117647 | 34 | 6 | 0.00009873 |
| *FLT1* | **---** | 0.44303797 | 32 | 7 | 0.00009253 |
| *HABP4* | **---** | 0.36777583 | 52 | 4 | 0.0000861 |
| *CAMK1* | **---** | 0.43209877 | 16 | 5 | 0.00008137 |
| *LDHD* | **---** | 0.33227848 | 76 | 10 | 0.00006271 |
| *DUSP1* | **---** | 0.45553145 | 54 | 8 | 0.00006268 |
| *NFKBIE* | **---** | 0.36206897 | 36 | 6 | 0.00005476 |
| *ENDOG* | **---** | 0.43209877 | 6 | 4 | 0.00003949 |
| *FOXM1* | **---** | 0.44117647 | 18 | 10 | 0.00003652 |
| *POLR2G* | **---** | 0.43298969 | 12 | 3 | 0.00003602 |
| *MMP13* | **---** | 0.32915361 | 6 | 3 | 0.00003087 |
| *MMP16* | **---** | 0.3271028 | 6 | 3 | 0.00002546 |
| *SUMO4* | **---** | 0.4312115 | 8 | 4 | 0.00002479 |
| *TNFSF10* | **---** | 0.37037037 | 30 | 13 | 0.00002382 |
| *IRAK3* | **---** | 0.33653846 | 8 | 8 | 0.00002312 |
| *MMP26* | **---** | 0.33175355 | 10 | 2 | 0.00001958 |
| *GSTA1* | **---** | 0.32915361 | 14 | 12 | 0.00000892 |
| *GSTA3* | **---** | 0.32915361 | 14 | 12 | 0.00000892 |
| *GSTA4* | **---** | 0.32915361 | 14 | 12 | 0.00000892 |
| *ME2* | **---** | 0.32307692 | 2 | 8 | 0.00000217 |
| *ME3* | **---** | 0.32307692 | 2 | 8 | 0.00000217 |
| *CHST4* | **---** | 0.24390244 | 0 | 1 | 0 |
| *CIDEA* | **---** | 0.23489933 | 0 | 1 | 0 |
| *ELN* | **---** | 0.27237354 | 0 | 1 | 0 |
| *IL10RB* | **---** | 0.33175355 | 0 | 3 | 0 |
| *MMP20* | **---** | 0.25893958 | 0 | 1 | 0 |
| *MMP24* | **---** | 0.26415094 | 0 | 1 | 0 |
| *MMP25* | **---** | 0.29871977 | 0 | 1 | 0 |
| *MPO* | **---** | 0.32110092 | 0 | 1 | 0 |
| *NFKBIZ* | **---** | 0.34653465 | 0 | 2 | 0 |
| *NOSIP* | **---** | 0.42857143 | 0 | 2 | 0 |
| *NOSTRIN* | **---** | 0.31626506 | 0 | 1 | 0 |
| *PAWR* | **---** | 0.43659044 | 0 | 4 | 0 |
| *PKLR* | **---** | 0.42682927 | 0 | 1 | 0 |
| *PPP3R2* | **---** | 0.28532609 | 0 | 1 | 0 |
| *SOD3* | **---** | 0.34257749 | 0 | 3 | 0 |
| *TFPI* | **---** | 0.30746706 | 0 | 4 | 0 |
| *TIMP4* | **---** | 0.33492823 | 0 | 2 | 0 |
| *TKTL1* | **---** | 0.4312115 | 0 | 2 | 0 |
| *TONSL* | **---** | 0.42944785 | 0 | 2 | 0 |
|  | | | | | |
| **AVERAGE** | | 0.4278415 | 2297.54 | 15.583 | 0.006697052 |
| + **1 SD** | | 0.492552 | 10277.8 | 29.4 | 0.042061202 |
| **+ 2 SD** | | 0.5572626 | 18258 | 43.217 | 0.077425351 |
